# A systematic literature review of randomized controlled trials evaluating prognosis following treatment for adults with chronic fatigue syndrome

**DOI:** 10.1017/S0033291722002471

**Published:** 2022-10

**Authors:** Tom Ingman, Abigail Smakowski, Kimberley Goldsmith, Trudie Chalder

**Affiliations:** 1 Department of Psychology, Institute of Psychiatry, Psychology and Neuroscience, Addiction Sciences Building, King's College London, London, UK; 2 Persistent Physical Symptoms Clinical Research and Treatment Unit, South London and Maudsley NHS Foundation Trust, London, UK; 3 Department of Biostatistics & Health Informatics, Institute of Psychiatry, Psychology and Neuroscience, King's College London, London, UK; 4 Department of Psychological Medicine, Institute of Psychiatry, Psychology and Neuroscience, King's College London, London, UK

**Keywords:** chronic fatigue syndrome, cognitive behavioral therapy, graded exercise therapy, literature review, prognosis

## Abstract

This systematic review investigated randomized controlled trials evaluating cognitive behavioral therapy (CBT) and graded exercise therapy (GET) for adults with chronic fatigue syndrome (CFS). The objective was to determine prognosis following treatment. Studies were eligible if they were peer-reviewed and investigated treatment at least 12 weeks in duration. Studies were excluded if they used co-morbid diagnoses as entry criteria or if they did not measure fatigue, disability, or functioning. Literature published between 1988 and 2021 was searched using MEDLINE, EMBASE, PsycINFO, and Web of Science. Study quality was assessed using the Effective Public Health Practice Project assessment tool. Outcomes were synthesized when three or more studies reported outcomes obtained from the same validated measurement tool. The review included 15 publications comprising 1990 participants. Following CBT, and at short-term to medium-term follow-up, 44% considered themselves better and 11% considered themselves worse. Following GET, and at post-treatment to short-term follow-up, 43% considered themselves better and 14% considered themselves worse. These outcomes were 8–26% more favorable compared to control conditions. Two-thirds of studies were of moderate quality and the remainder were of weak quality. Limitations of this review relate to the clinical heterogeneity of studies and that most outcomes were self-reported. Results suggest some support for the positive effects of CBT and GET at short-term to medium-term follow-up although this requires further investigation given the inconsistent findings of previous reviews. Findings may not be generalizable to severe CFS. This review was registered with PROSPERO (CRD42018086002).

## Introduction

Chronic fatigue syndrome (CFS) is a serious illness characterized by persistent and medically unexplained fatigue which is severe enough to result in substantial disability. Other symptoms such as musculoskeletal pain, sleep disturbance, and cognitive dysfunction are common (Collin et al., 2016). Some researchers consider myalgic encephalomyelitis (ME) to be the same disorder, while others consider it a different condition with separate diagnostic criteria (Lim & Son, 2020). The current review uses the term CFS, rather than ME, as this has been operationalized in the literature. The National Institute for Health and Care Excellence (NICE, 2007) estimates the prevalence of CFS in the UK to be 0.2–0.4%.

Prognosis, which is defined as the proportion of subjects who improve or worsen according to a specific outcome, and within a discrete time period, plays a critical role in bridging the gap between research and practice (Kent, Cancelliere, Boyle, Cassidy, & Kongsted, 2020; Moons et al., 2018). Indeed, the percentage change, not mean change, may be the most relevant determinant of outcome in CFS (Schluederberg et al., 1992). In their review of naturalistic, cohort, and intervention studies, Cairns and Hotopf (2005) reported that over time, 39% of patients with CFS showed some improvement, 7% recovered, and 5–20% deteriorated.

Prognosis following treatment is less clear, partly because few systematic reviews have reported it and partly because there is no gold standard treatment. Nonetheless, current literature shows that cognitive behavioral therapy (CBT) and graded exercise therapy (GET) are the most promising treatments, both of which yield improvements in fatigue and functioning (Castell, Kazantzis, & Moss-Morris, 2011; Larun, Brurberg, Odgaard-Jensen, & Price, 2019; Malouff, Thorsteinsson, Rooke, Bhullar, & Schutte, 2008; Marques, De Gucht, Gouveia, Leal, & Maes, 2015; Price, Mitchell, Tidy, & Hunot, 2008). One review reported that, following CBT, between 40% and 48% of patients showed clinical improvements in fatigue, compared to 26% receiving usual care and 27% receiving other therapies, such as relaxation (Price et al., 2008). In another review, Malouff et al. (2008) reported that, at follow-up, 50% of patients receiving CBT were within the ‘normal range’ on a variety of outcomes.

However, reviews such as these are limited by the synthesis of data obtained from multiple measurement tools, the inclusion of non-randomized studies, which may produce between-group imbalances or biased findings, or studies with a short-term follow-up. The use of non-specialist diagnostic procedures, or broad inclusion criteria (e.g. idiopathic chronic fatigue), may also lead to misclassification and yield findings that do not apply to CFS (Newton, Mabillard, Scott, Hoad, & Spickett, 2010). Further research reviewing randomized treatment trials which employ stricter diagnostic criteria, utilize the same outcome measures, and have a longer follow-up, is therefore required to determine prognosis following treatment.

### Aims

This review aimed to assess randomized controlled trials investigating CBT and GET, compared to active or passive control conditions, to extract dichotomous outcomes, and determine the prognosis of CFS in an adult population.

## Method

### Design

A systematic review (PROSPERO registration: CRD42018086002) was conducted following PRISMA guidelines (Moher, Liberati, Tetzlaff, & Altman, 2009). Ethical approval was not required for a systematic review. A person with lived experience of CFS contributed to this research.

### Study selection criteria

Studies were eligible if they: (1) contained original data from an English-language, peer-review journal; (2) were randomized and controlled; (3) included participants who met at least one case definition for CFS, including the Oxford criteria (Sharpe et al., 1991) which require 6 months of severe fatigue affecting physical and mental functioning, is present over 50% of the time, and is accompanied by symptoms such as myalgia, mood, and sleep disturbance; CDC criteria (Fukuda et al., 1994) which require 6 months of persistent or relapsing fatigue, substantially reduced functioning, and at least four additional symptoms (e.g. sleep disturbance, sore throat, headaches, or post-exercise malaise); or NICE criteria (NICE, 2007), which requires 4 months of persistent or relapsing fatigue, substantially reduced activity, post-exertional malaise and/or fatigue, and at least one of ten symptoms, including those listed above. Criteria stipulating that screening or assessment was conducted by a secondary care medical doctor or psychiatrist was added during screening to increase the probability of an accurate diagnosis; (4) investigated individually or group-based CBT or GET, or sufficient components of these treatments (e.g. for CBT, addressing perpetuating cognitive and behavioral factors, and increasing activity, and for GET, incrementally increasing physical exercise) as well as these treatments in combination with other interventions not routinely applied (e.g. pharmacotherapy when needed), with treatment at least 12 weeks in duration; and (5) described treatment outcome. Active controls (psycho-social, psychological, and/or pharmacological treatments), passive controls (specialist medical care, relaxation, or flexibility treatments), and non-inferiority studies comparing different CBT or GET protocols were included. There was no maximum follow-up period or upper age limit for inclusion.

Studies were excluded if they: (1) included participants <18 years old; (2) used idiopathic chronic fatigue or co-morbid diagnoses as entry criteria; (3) did not include at least one outcome related to fatigue, disability, functioning, or quality of life; and (4) were economic evaluations, ecological or case-control studies, or cross-over trials without an independent control group.

### Databases and search strategy

Published literature was searched using MEDLINE, EMBASE, PsycINFO, and Web of Science, between 1988, the year of first case definition of CFS, and March 2021. Other databases listed in the PROSPERO protocol were not used as four were considered sufficient. The search was performed using the PICO (patient/intervention/comparator/outcome) model with keywords such as chronic fatigue syndrome, cognitive behavioral or graded exercise therapy, randomized controlled trials and prognosis combined with the ‘AND’ Boolean operator. Searches in MEDLINE, EMBASE, and PsycINFO were limited to human studies and searches in Web of Science excluded studies of children or adolescents. Online Supplementary eAppendix 1 shows the search strategy for each database.

### Study selection

Authors TI and AS independently reviewed all titles and abstracts for eligibility using the Rayyan citation website (Ouzzani, Hammady, Fedorowicz, & Elmagarmid, 2016), in addition to retrieving and reviewing full texts for papers that met inclusion criteria or those in which eligibility was unclear. TI and AS were blinded to each other's review and disagreements regarding eligibility were resolved by discussion.

### Terminology and definitions

Prognosis referred to dichotomous outcomes relating to the proportion of subjects who had improved or worsened according to a specific outcome (e.g. fatigue), and during a discrete time period following treatment. Outcomes were reported at various time-points and categorized as post-treatment, short-term follow-up (1–6 months post-treatment), medium-term follow-up (7–12 months post-treatment), and long-term follow-up (>12 months post-treatment).

### Data extraction

Extracted data were entered onto a Microsoft Excel (Version 16) spreadsheet by TI and checked for accuracy and completeness by AS. The data extraction sheet template can be found in online Supplementary eAppendix 2. To increase validity and focus, outcomes obtained before or after the primary outcome point, which concerned participant subsets, or which were measured using unvalidated measurement tools, and predictive factors, were not extracted. Fulcher and White (1997) was a cross-over trial and findings following cross-over were not extracted. To maximize the reliability of outcomes obtained from the global impression-improvement scale (CGI-I; Guy, 1976), outcomes from one study (Jason et al., 2007) which defined improvement as ‘better’, ‘much better’, or ‘very much better’, without describing the raw data to allow comparison with other studies, were not reported. Two studies (Clark et al., 2017; Jason et al., 2007) used a variant of the CGI-I (e.g. a 6-point scale), so were also excluded. Adverse effects reported by three studies (Clark et al., 2017; Janse, Worm-Smeitink, Bleijenberg, Donders, & Knoop, 2018; White et al., 2011) were not extracted, as deterioration or exacerbation, also reported by these studies, were considered more relevant to prognosis at follow-up. Additional data related to participant worsening on the CGI-I were obtained from authors of three papers (Clark et al., 2017; Moss-Morris, Sharon, Tobin, & Baldi, 2005; White et al., 2011) and used in the synthesis reported in Table 4. A summary of this data can be found in Tables 2 and 3 (see superscripts) and a further breakdown is available on request from authors of the current review.

### Study quality and risk of bias

Study quality was assessed using the Effective Public Health Practice Project (EPHPP) quality assessment tool for quantitative studies (Thomas, Ciliska, Dobbins, & Micucci, 2004). This tool was chosen because it has content and construct validity, assesses the psychometrics of data collection tools, and may be more reliable than other tools (Armijo-Olivo, Stiles, Hagen, Biondo, & Cummings, 2012; Minozzi, Cinquini, Gianola, Gonzalez-Lorenzo, & Banzi, 2020). The EPHPP comprises six domains (see online Supplementary eAppendix 4) from which a global rating can be calculated. Studies were penalized for confounding if there were clinically important between-group differences in baseline scores of a main outcome (e.g. fatigue or physical functioning). Studies were coded as ‘strong’ (no weak ratings), ‘moderate’ (one weak rating), or ‘weak’ (two or more weak ratings). Studies were independently rated (TI and AS) and disagreements were resolved by consensus. The EPHPP authors were also contacted to clarify that studies should not be penalized for lacking individual reasons for participant withdrawal (D. Ciliska, personal communication, 15 April 2022), which resulted in five studies originally rated as ‘poor’ to be re-rated as ‘moderate’ or ‘strong’ on this outcome (Fulcher & White, 1997; Powell, Bentall, Nye, & Edwards, 2001; White et al., 2011; White, Goldsmith, Johnson, Chalder, & Sharpe, 2013; Wiborg, van Bussel, van Dijk, Bleijenberg, & Knoop, 2015).

### Statistical analysis and data synthesis

The following is an update and extension of the strategy described in the PROSPERO protocol, which was based on a previous review of naturalistic, cohort, and intervention studies reporting prognosis in CFS (Cairns & Hotopf, 2005). It incorporates methods used in reviews of intervention-based trials (Malouff et al., 2008; Price et al., 2008). Data were analyzed using Microsoft Excel (Version 16) and IBM SPSS Statistics for Macintosh (version 26). Means (and ranges) were used to summarize participant age and treatment follow-up and medians (and ranges) to summarize number of treatment sessions and treatment duration. Descriptive statistics (percentages) were used to report variables related to participant and study characteristics such as gender, diagnostic criteria, type of control condition, and prognostic outcomes for individual studies. Synthesis was conducted when three or more studies reported outcomes obtained from the same measurement tool. Synthesis of participant improvement involved summing the total number of participants who improved in the relevant (CBT or GET) intervention groups in the selected studies divided by the total number randomized to these conditions to produce a weighted mean percentage. This procedure assumed that participants without outcome data were non-improvers. The same procedure was conducted for improvers in non-CBT or non-GET control conditions. Synthesis of participant worsening involved dividing the number of participants who had worsened by the number of outcome completers only, rather than all those randomized, to avoid underestimating this outcome. Differences in prognosis between intervention and control conditions for each synthesized outcome were calculated by subtracting one from the other and 95% confidence intervals (CIs) for these differences were calculated. Post-hoc sensitivity analysis of global worsening was conducted assuming that those without outcome data had worsened. To assess the robustness of findings, additional analyses were conducted which excluded data from studies of weak quality thereby generating estimates of prognosis based on studies of at least moderate quality. Meta-analyses were not conducted as it was not clear how many outcomes would fulfill criteria for pooling data and therefore it was not possible to specify primary outcomes *a-priori*. Conducting post-hoc meta-analyses may have increased the chance of multiple testing and reporting biases. Furthermore, several previous reviews have assessed the efficacy of CBT and GET using meta-analytic methods (Castell et al., 2011; Larun et al., 2019; Malouff et al., 2008; Marques et al., 2015; Price et al., 2008).

### Unit of analysis

The intervention and control arms of studies investigating one CBT or GET condition were compared. Data were pooled when intervention arms in the same study had more than one CBT or GET condition (i.e. Janse et al. 2018; Powell et al. 2001; Wiborg et al. 2015; Worm-Smeitink et al. 2019) and when both the intervention and control in the same study comprised different CBT conditions (i.e. Tummers, Knoop, & Bleijenberg, 2010; Worm-Smeitink et al. 2019).

## Results

### Description of included studies

**Fig. 1.**
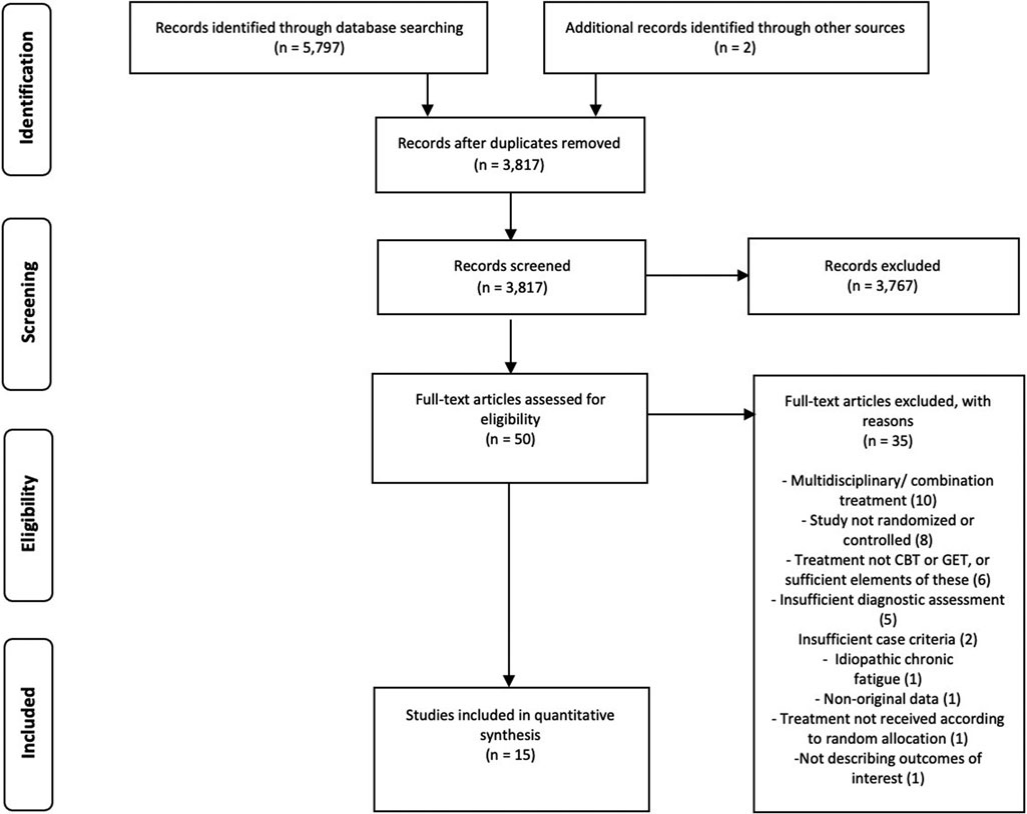
PRISMA flowchart of study selection.

After removing duplicates, 3817 studies were identified. After screening, 3767 (99%) were excluded, resulting in 50 full texts. Reasons for exclusion and references to excluded studies can be found in online Supplementary eAppendix 3. Reviewing full texts led to further exclusion of 35 studies, resulting in 15 included publications. White et al. (2011, 2013) reported from the same trial which resulted in 11 publications with a CBT condition (see Table 2), reporting from ten trials, and six studies with a GET condition (see Table 3), reporting from five trials. Figure 1 shows a flow diagram of the selection process.

### Reliability of study selection

Reviewers were initially discrepant on 32 (1%) occasions when screening titles and abstracts for study eligibility. These discrepancies were resolved through discussion.

### Study characteristics

Study characteristics are shown in Tables 2 and 3. Studies were conducted in the UK (*n* = 7), the Netherlands (*n* = 6), the USA (*n* = 1), and New Zealand (*n* = 1).

### Participant characteristics

Eligible studies comprised 1990 participants, excluding 1266 reported in another included study or in an excluded treatment arm. As seen in Tables 2 and 3, a diagnosis of CFS was made using the CDC criteria (*n* = 8, 50%), the Oxford criteria (*n* = 5, 31%), both the CDC and the Oxford criteria (*n* = 1, 7%), or the NICE (2007) criteria (*n* = 1, 6%).

### Characteristics of CBT

As shown in Table 2, treatment in six of the ten trials with a CBT condition was face-to-face. Of the remaining four trials, one (Janse et al., 2018) comprised internet-based CBT with higher (fortnightly therapist contact) and lower resource conditions (therapist contact when requested), and one (Knoop, van der Meer, & Bleijenberg, 2008) consisted of self-help manuals; clinician contact in both was mostly remote (e.g. via telephone). Tummers et al. (2010) and Worm-Smeitink et al. (2019) investigated a stepped-care approach incorporating the treatment protocol in Knoop et al. (2008) and Janse et al. (2018), respectively, followed by face-to-face CBT if required. The control condition in Tummers et al. (2010) and Worm-Smeitink et al. (2019) comprised face-to-face CBT.

Participants were offered a median of 14 sessions (range: 13–16) in eight trials with a face-to-face CBT condition. Participants in White et al. (2011, 2013) were offered a follow-up CBT session and received a median of three specialist medical care sessions. Participants in Sharpe et al. (1996) were offered medical care consultations (frequency not reported). Participants in the higher resource Internet-based cognitive behavioral therapy (iCBT) condition in Janse et al. (2018) were instructed to contact their therapist 12 times. The self-help CBT in Knoop et al. (2008) required participants to contact their therapist every 2 weeks over a 16-week period. The median duration of treatment offered in CBT conditions was 26 weeks (range: 16–52).

### Characteristic of GET

Table 3 shows that three trials (Fulcher & White, 1997; Moss-Morris et al., 2005; White et al., 2011; 2013) investigated face-to-face GET, with a median of 12 sessions offered (range: 12–14). Participants in White et al. (2011, 2013) were offered a follow-up session and received a median of three specialist medical care sessions. Clark et al. (2017) investigated a 12-week self-help graded exercise treatment comprising four guidance sessions, delivered face-to-face or remotely, plus ⩾1 specialist medical care consultation. Powell et al. (2001) investigated various ‘doses’ of education to encourage a self-managed graded exercise program, comprising between three and ten sessions, delivered face-to-face or remotely, with additional consultations available on request. The median duration of GET treatment was 12 weeks (range: 12–23).

### Characteristics of (non-CBT/GET) comparison conditions

The control condition in five trials (Clark et al., 2017; Moss-Morris et al., 2005; Powell et al., 2001; Sharpe et al., 1996; White et al., 2011; 2013) comprised ⩾1 sessions of specialist or standard medical care (e.g. generic advice or medication), in three trials (Deale, Chalder, Marks, & Wessely, 1997; Fulcher & White, 1997; Jason et al., 2007) comprised relaxation or flexibility treatment (between ten and 13 sessions offered), and in three trials (Janse et al., 2018; Knoop et al., 2008; Wiborg et al., 2015) consisted of a wait list control. The control in one trial (Vos-Vromans et al., 2016) was multidisciplinary rehabilitation treatment, comprising 44.5 h of CBT, body awareness therapy, pacing, mindfulness, social reintegration, and sleep normalization, delivered by a multidisciplinary team of clinicians.

### Outcome equivalence in pooled treatment conditions

There were no significant differences between pooled conditions in primary and secondary outcomes (e.g. fatigue and physical functioning) in trials comparing higher and lower resource or intensity CBT (Janse et al., 2018; Tummers, Knoop, van Dam, & Bleijenberg, 2013; Wiborg et al., 2015; Worm-Smeitink et al., 2019) or higher and lower intensity GET (Powell et al., 2001)

### Outcome measures

Table 1 lists the outcome measures and cut-off scores used by each included study. Fatigue was an outcome in nine studies, with six of these (Janse et al., 2018; Knoop et al., 2008; Tummers et al., 2010; Vos-Vromans et al., 2016; Wiborg et al., 2015; Worm-Smeitink et al., 2019) using the self-report Checklist of Individual Strength (CIS; Vercoulen et al., 1994; range: 8–56; higher = more fatigue) and three (Clark et al. 2017; Deale et al. 1997; White et al. 2011) using the 11-item self-report Chalder Fatigue Questionnaire (CFQ; Chalder et al., 1993) which can be scored bimodally (range: 0–11) or on a Likert scale (range: 0–33), with higher scores indicating more fatigue.

**Table 1.** Criteria for prognostic outcomes following treatment

Reference	Outcome	Criteria
Clark et al. (2017)	Global improvement Clinically significant improvement in fatigue and physical functioning Increase in physical activity Global worsening^a^ Serious deterioration	CGI-I ratings of ‘much better’ or ‘very much better’ ⩾3-point Likert score decrease on the 11-item CFQ and an ⩾8-point increase on the SF-36 physical functioning subscale High level of activity in the previous week according to the IPAQ CGI-I ratings of ‘worse’, ‘much worse’, or ‘very much worse’ ⩾10-point reduction on the SF-36 physical functioning subscale, CGI-I ratings of ‘much worse’ or ‘very much worse’, or active withdrawal due to worsening
Deale et al. (1997)	Global improvement Clinically significant improvement in physical functioning No longer fatigue case No longer psychological case (mood and distress) No longer meeting CFS criteria Global worsening^a^	CGI-I ratings of ‘much better’ or ‘very much better’ Score of ⩾83 or a ⩾50-point increase on the SF-36 physical functioning subscale Bimodal scores of <4 on the 11-item CFQ Score of <4 on the GHQ Oxford and CDC criteria CGI-I ratings of ‘a little worse’, ‘much worse’, or ‘very much worse’
Fulcher and White (1997)	Global improvement Global worsening	CGI-I ratings of ‘much better’ or ‘very much better’ CGI-I ratings of ‘a little worse’, ‘much worse’, or ‘very much worse’
Janse et al. (2018)	Normal range for fatigue Exacerbation in fatigue, physical functioning, disability, and distress	RCI >1.96 and a score of <35 on the CIS fatigue subscale RCI of <−1.96 on the CIS fatigue subscale, SF-36 physical functioning subscale, SIP8, and SCL-90
Jason et al. (2007)	Clinically significant improvement in physical functioning No longer meeting CFS criteria Improved work status	Change score exceeds age-adjusted RCI on the SF-36 physical functioning subscale and follow-up scores within 1 s.d. of the normative value^b^ CDC criteria Working ⩾20 h per week or in full-time education
Knoop et al. (2008)	Clinically significant improvement in fatigue	RCI >1.96 and a score of <35 on the CIS fatigue subscale
Moss-Morris et al. (2005)	Global improvement Global worsening^a^	CGI-I ratings of ‘much better’ or ‘very much better’ CGI-I ratings of ‘a little worse’, ‘much worse’, or ‘very much worse’
Powell et al. (2001)	Global improvement Clinically significant improvement in physical functioning	CGI-I ratings of ‘much better’ or ‘very much better’ Score of ⩾25 or a ⩾10-point increase on the SF-36 physical functioning subscale^c^
Sharpe et al. (1996)	Global improvement Clinically significant improvement in functioning Normal range for functioning Improved work status Global worsening	CGI-I ratings of ‘much better’ or ‘very much better’ ⩾10-point increase on the Karnofsky scale Score of ⩾80 on the Karnofsky scale Working or studying CGI-I ratings of ‘a little worse’, ‘much worse’, or ‘very much worse’
Tummers et al. (2010)	Clinically significant improvement in fatigue	RCI >1.96 and a score of <35 on the CIS fatigue subscale
Vos-Vromans et al. (2016)	Clinically significant improvement in fatigue	Score of <35 on the CIS fatigue subscale
Wiborg et al. (2015)	Clinically significant improvement in fatigue, physical functioning, and disability Recovery	RCI >1.96 and a score of <35 on the CIS fatigue subscale, score of ⩾65 on the SF-36 physical functioning subscale, and <700 on the SIP-8 Score of <27 on the CIS fatigue subscale, ⩾80 on the SF-36 physical functioning subscale, and <203 on the SIP-8
White et al. (2011)	Global improvement Clinically significant improvement in fatigue and physical functioning Normal range for fatigue and physical functioning Global worsening^a^ Serious deterioration	CGI-I ratings of ‘much better’ or ‘very much better’ ⩾2-point Likert score decrease on the 11-item CFQ and an ⩾8-point increase on the SF-36 physical functioning subscale Likert score of ⩽18 on the 11-item CFQ and ⩾60 on the SF-36 physical functioning subscale CGI-I ratings of ‘a little worse’, ‘much worse’, or ‘very much worse’ A decrease (between baseline and two consecutive assessments) of ⩾20 on the SF-36 physical function subscale, CGI-I ratings of ‘much worse’ or ‘very much worse’ at two consecutive assessments, withdrawal after 8 weeks because of feeling worse, or a serious adverse reaction (determined by a masked clinician)
White et al. (2013)	Recovery	Likert score of ⩽18 on the 11-item CFQ, ⩾60 on the SF-36 physical functioning subscale, not meeting Oxford criteria, and CGI-I ratings of ‘much better’ or ‘very much better’
Worm-Smeitink et al. (2019)	Clinically significant improvement in fatigue	RCI >1.96 and a score of <35 on the CIS fatigue subscale

*Note*. CGI-I, Clinical Global Impressions-Improvement scale; CFQ, Chalder Fatigue Questionnaire; SF-36, Short-Form Health Survey-36; IPAQ, International Physical Activity Questionnaire; GHQ, General Health Questionnaire; CDC, Centre for Disease Control and Prevention; RCI, reliable change index; CIS, Checklist of Individual Strength; SIP8, Sickness Impact Profile; SCL-90, Symptom Checklist 90; s.d., standard deviation.

a Criteria applied in this review but not used by study authors.

b Not reported by study authors.

c Reported on a 10–30 scale; when converted to the more common 0–100 scale by using the following formula: mean_new_ = (mean_old_–10) × 5, a score of 25 = 75 and a 10-point increase = 50 points.

Physical functioning was an outcome in seven studies, all of which used the self-rated Short-Form Health Survey (SF-36) physical functioning subscale (Ware & Sherbourne, 1992). This measures limitations in activities such as ‘walking 100 yards’ (range: 0–100; higher = less limited). General functioning was an outcome in one study (Sharpe et al., 1996) and was measured using the clinician-rated Karnofsky scale (Karnofsky, Abelmann, Craver, & Burchenal, 1948) following an interview with the participant (and cohabitee when possible) about their activities over the previous month (range: 0–100; higher = less limited).

Global change in health was an outcome in six studies, all of which used the self-rated Clinical Global Impression-Improvement scale (CGI-I; Guy, 1976). This asks respondents to rate change on a 7-point scale from ‘very much better’ to ‘very much worse’ compared to study onset. Authors of studies listed in Table 1 defined improvement as ‘much better’ or ‘very much better’. Study authors defined worsening using various combinations of responses, therefore to maximize available data, ratings of ‘a little worse’, ‘much worse’, and ‘very much worse’ were combined and reported in the current review as ‘global worsening’.

Other prognostic outcomes included disability, mental distress, levels of activity, and employment. Composite measures were used to define ‘recovery’ in two studies (White et al., 2011, 2013) and ‘deterioration’ in a further two studies (Clark et al., 2017; White et al., 2011). Primary outcomes, as defined by study authors, are described in Tables 2 and 3. The abovementioned measurement tools are valid and reliable, except for the reliability of the CGI-I, which is difficult to ascertain given that it captures self-reported change relative to pre-treatment.

### Study quality and risk of bias

The quality rating of ten studies (Clark et al., 2017; Deale et al., 1997; Fulcher & White, 1997; Moss-Morris et al., 2005; Powell et al., 2001; Sharpe et al., 1996; Vos-Vromans et al., 2016; White et al., 2011; 2013; Wiborg et al., 2015) was ‘moderate’ and the remainder ‘weak’ (Tables 2 and 3). Ratings by domain can be seen in online Supplementary eAppendix 4. All studies were penalized for the lack of blinding and five (33%) for not adequately describing drop-out per study group. Jason et al. (2007) was also penalized for confounding given the potential for clinically significant between-group differences in baseline scores of physical functioning which were not controlled for in the (pre-specified) analysis or discussed satisfactorily by authors.

### Main findings

#### Prognosis following CBT

Table 2 shows all outcomes following CBT. Table 4 shows that four outcomes, obtained using three measurement tools, were included in the synthesis: (1) significant improvements in fatigue (at post-treatment to short-term follow-up); (2) significant improvements in physical functioning (at short-term to medium-term follow-up); (3) global improvements (at short-term to medium-term follow-up); and (4) global worsening (at short-term to medium-term follow-up). The synthesis included data from nine (90%) CBT trials. Table 1 shows that the same criteria were used for calculating global improvements and global worsening, but not for calculating improvements in physical functioning. The same cut-off scores were used to calculate improvements in fatigue, however, all but one study (Vos-Vromans et al., 2016) used the reliable change index as additional criteria when calculated this outcome.

**Table 2.** Characteristics of included CBT studies, their participants, and prognosis

Reference (author/year)	Setting and diagnostic criteria	Intervention and control	Sample size Intervention (#) Control (#)	Population: age (mean years); gender (% female)	Primary outcome and follow-up	Prognosis following CBT treatment(s) (% of participants)	EPHPP global quality rating
Deale et al. (1997)	CFS clinic, UK; Oxford and CDC Criteria	CBT *v*. relaxation	Intervention: 30 Control: 30	Intervention: 31; 70 Control: 38; 67	Physical functioning; short-term follow-up	63% globally improved; 63% significantly improved in physical functioning; 43% improved in mood; 57% no longer fatigue cases; 50% no longer met CFS criteria; 0% globally worsened	Moderate
Janse et al. (2018)	CFS Clinic, Netherlands; CDC Criteria	iCBT with feedback (i) or iCBT with feedback on demand only (ii) *v*. WL	Intervention: 80 in both conditions Control: 80	iCBT (i) 37; 68. iCBT (ii) 36; 58 Control: 40; 56	Fatigue; post-treatment	39% in normal range for fatigue; 5% experienced exacerbation in fatigue, 3% in impairment, 5% in physical functioning, and 9% in psychological distress	Weak
Jason et al. (2007)	Hospital, USA; CDC Criteria	CBT *v*. relaxation	Intervention: 29 Control: 28	Entire sample: 44; 83	None stated; medium-term follow-up	18%^a^ significantly improved in physical functioning; 14% no longer met CFS criteria; 17%^a^ improved in work status	Weak
Knoop et al. (2008)	CFS clinic, Netherlands; CDC criteria	Guided self-instruction CBT *v*. WL	Intervention: 84^b^ Control: 85^b^	See Tummers et al. (2010)	Fatigue, physical functioning, and disability; post-treatment	27% significant improved in fatigue	Weak
Sharpe et al. (1996)	Infectious disease outpatient clinic, UK; Oxford criteria	CBT *v*. StMC	Intervention 30 Control: 30	Intervention: 34; 60 Control: 38; 77	Functioning; medium-term follow-up	60% globally improved; 73% significantly improved in physical functioning; 73% in normal range for physical functioning; 63% improved in work status; 7% deteriorated and attributed this to treatment; 13% globally worsened	Moderate
Tummers et al. (2010)	CFS clinic, Netherlands; CDC criteria	Self-help CBT *v*. WL (both conditions followed by face-to-face CBT if needed)	Intervention: 84^b^ Control: 85^b^	Intervention: 38; 82 Control: 39; 75	Fatigue, physical functioning, and disability; post-treatment	48% significantly improved in fatigue	Weak
Vos-Vromans et al. (2016)	Rehabilitation center, Netherlands; CDC Criteria	MRT *v*. CBT	Intervention: 62 Control: 60	Intervention: 40; 81 Control: 41; 78	Fatigue and health-related quality of life; short-term follow-up	26% significantly improved in fatigue	Moderate
Wiborg et al. (2015)	CFS outpatient clinic, Netherlands; CDC criteria	CBT group therapy: large CBT group (i) and small CBT group (ii) *v*. WL	Intervention: CBT (i): 68 CBT (ii): 68 Control: 68	Intervention: CBT (i) 36; 75 CBT (ii) 40; 74 Control: 37; 74	Fatigue, physical functioning and disability; post-treatment	49% significantly improved in fatigue and 38% in all primary outcome measures; 15% recovered	Moderate
White et al. (2011)	Secondary care clinics, UK; Oxford Criteria	CBT *v*. SpMC	Intervention: 161 Control: 160	Intervention: 39; 80 Control: 37; 76	Fatigue and physical functioning; short-term follow-up	38% globally improved; 70% significantly improved in fatigue and 65% in physical functioning; 27% in normal ranges for fatigue and physical functioning; 13%^c^ globally worsened; 9% seriously deteriorated	Moderate
White et al. (2013)	Secondary care clinics, UK; Oxford Criteria	CBT *v*. SpMC	Intervention: 161 Control: 160	Entire sample: 38; 77	Fatigue and physical functioning; short-term follow-up	20% recovered	Moderate
Worm-Smeitink et al. (2019)	CFS clinic, Netherlands; CDC criteria	iCBT with feedback (i) or iCBT with feedback on demand only (ii), *v*. WL (all conditions followed by face-to-face CBT if needed)	Intervention: 121 in both conditions Control: 121	iCBT (i) 37; 64 iCBT (ii) 37; 57 Control 39; 61	Fatigue; post-treatment	45% significantly improved in fatigue	Weak

*Note*. EPHPP, Effective Public Health Practice Project; CFS, chronic fatigue syndrome; CDC, Centre for Disease Control and Prevention; CBT, cognitive behavioral therapy; iCBT, Internet-based cognitive behavioral therapy; WL, waiting list; StMC, standard medical care; MRT, multidisciplinary rehabilitation treatment; SpMC, specialist medical care.

a Study authors did not report if outcomes were based on all participants randomized to treatment, or a subset who completed outcomes only.

b Following randomization, one participant from the intervention and one from the control condition were excluded due to medical explanations for their fatigue.

c Data obtained from study author.

Table 4 shows that the proportion of improved participants was higher following CBT, compared to control, for all synthesized outcomes: 23% (95% CI 16–31) more participants improved in fatigue, 14% (95% CI 4–23) more improved in physical functioning, and 20% (95% CI 11–29) more rated themselves as improved. Table 4 also shows that 8% (95% CI 1–14) fewer participants reported global worsening following CBT compared to control. Global worsening following CBT remained lower than in the control condition when it was assumed that participants lost to follow-up had also worsened (18% *v.* 23%, respectively).

When excluding three studies of weak quality used in the synthesis of fatigue (Knoop et al., 2008; Tummers et al., 2010; Worm-Smeitink et al., 2019), the proportion of participants who showed significant improvement was 42% following CBT and 28% in the control group (14% difference; 95% CI 0–24), 1% lower and 8% higher compared to the original analysis, respectively. When excluding one study of weak quality used in the synthesis of physical functioning (Jason et al., 2007), the proportion of participants who showed significant improvement was 65% following CBT and 49% in the control group (16% difference; 95% CI 1–26), 6% and 4% higher than in the original analysis, respectively. All data used in the synthesis of global improvement and global worsening were drawn from studies of moderate quality.

#### Prognosis following GET

Table 3 shows all outcomes following GET. Table 4 shows that three outcomes, obtained using two outcome measures, were included in the data synthesis: (1) significant improvement in physical functioning (at post-treatment to medium-term follow-up); (2) global improvement (at post-treatment to short-term follow-up); and (3) global worsening (at post-treatment to short-term follow-up). All five GET trials contributed data to synthesis of these outcomes. Table 1 shows that the same criteria were used for outcomes except for physical functioning.

**Table 3.** Characteristics of included GET studies, their participants, and prognosis

Reference (author/year)	Setting and diagnostic criteria	Intervention and control	Sample size Intervention (#) Control (#)	Population characteristics: age (mean years); gender (% female)	Primary outcome	Prognosis following GET treatment(s) (% of participants)	EPHPP global quality rating
Clark et al. (2017)	CFS clinic, UK; NICE criteria	Self-help GET *v*. SpMC	Intervention: 107 Control: 104	Intervention: 38; 82 Control: 39; 76	Fatigue and physical functioning; post-treatment	16% globally improved; 58% significantly improved in fatigue and 41% in physical functioning; 24% increased physical activity; 21% seriously deteriorated; 19%^a^ globally worsened	Moderate
Fulcher and White (1997)	CFS clinic, UK; Oxford Criteria	GET *v*. flexibility and relaxation	Intervention: 33 Control: 33	Entire sample: 37; 74	Global improvement; post-treatment	52% globally improved; 3% globally worsened	Moderate
Moss-Morris et al. (2005)	CFS private general practice, New Zealand; CDC Criteria	GET *v*. StMC	Intervention: 25 Control: 24	Intervention: 37; 60. Control: 46; 79	Global improvement; post-treatment	48% globally improved; 18%^a^ globally worsened	Moderate
Powell et al. (2001)	CFS clinic and an infectious disease outpatient clinic, UK; Oxford criteria	‘Doses’ of education to encourage GET: minimum (i), telephone (ii), and maximum (iii) conditions *v*. StMC	Intervention: GET (i) 37 GET (ii) 39 GET (iii) 38 Control: 34	Intervention GET (i) 34; 76 GET (ii) 32; 85 GET (iii) 33; 82 Control: 34; 71	Fatigue and physical functioning; medium-term follow-up	70% globally improved; 69% significantly improved in physical functioning	Moderate
White et al. (2011)	Secondary care clinic, UK; Oxford Criteria	GET *v*. SpMC	Intervention: 160 Control: 160	Intervention: 39; 77. Control: 37; 76	Fatigue and physical functioning; short-term follow-up	39% globally improved; 77% significantly improved in fatigue and 68% in physical functioning; 27% in normal ranges for fatigue and physical functioning; 13%^a^ globally worsened; 6% seriously deteriorated	Moderate
White et al. (2013)	Secondary care clinics, UK; Oxford Criteria	GET *v*. SpMC	Intervention: 160 Control: 160	Entire sample: 38; 77	Fatigue and physical functioning; short-term follow-up	20% recovered	Moderate

*Note*. EPHPP, Effective Public Health Practice Project; CFS, chronic fatigue syndrome; GET, graded exercise therapy; StMC, standard medical care; NICE, National Institute for Health and Clinical Excellence; SpMC, specialist medical care; CDC, Centre for Disease Control and Prevention.

a Data obtained from study author.

Table 4 shows that the proportion of improved participants was higher following GET, compared to control, for both synthesized outcomes: 23% (95% CI 15–31) more participants receiving GET significantly improved in physical functioning compared to those in the control and 26% (95% CI 19–32) more reported global improvements. Table 4 also shows that 10% (95% CI 4–16) fewer participants reported global worsening following GET compared to the control condition. Global worsening following GET remained lower than in the control condition when participants lost to follow-up were assumed to have worsened (21% *v.* 28%, respectively). All data used in the synthesis of each of the above outcomes were drawn from GET studies of moderate quality.

**Table 4.** Prognosis following CBT, GET, and control conditions

Outcome	CBT			GET		
	Study (*N*)	Intervention	Control^a^	Study (*N*)	Intervention	Control
Significant improvement in fatigue severity^b^	5^1–5^	43 (26–49)	20 (7–49)	–	–	–
Significant improvement in physical functioning^c^	3^6–8^	59 (18–65)	45 (17–55)	3^6,10,11^	61 (41–69)	38 (6–55)
Global improvement^d^	3^6,7,9^	44 (38–63)	24 (23–27)	5^6,10–13^	43 (16–70)	17 (5–27)
Global worsening^e^	3^6,7,9^	11 (0–13)	19 (10–20)	4^6,10,12,13^	14 (3–19)	24 (3–35)

*Note*. Data are mean percentages and ranges unless specified otherwise.

1: Knoop et al. (2008); 2: Tummers et al. (2010); 3: Wiborg et al. (2015); 4: Worm-Smeitink et al. (2019); 5: Vos-Vromans et al. (2016); 6: White et al. (2011); 7: Deale et al. (1997); 8: Jason et al. (2007); 9: Sharpe et al. (1996); 10: Clark et al. (2017); 11: Powell et al. (2001); 12: Moss-Morris et al. (2005); 13: Fulcher and White (1997).

a Excludes conditions in which the control was also CBT.

b Measured using the Checklist of Individual Strength.

c Measured using the Short-Form Health Survey-36 physical functioning subscale.

d Ratings of ‘much better’ or ‘very much better’ on the Clinical Global Impressions-Improvement scale.

e Ratings of ‘a little worse’, ‘much worse’, or ‘very much worse’ on the Clinical Global Impressions-Improvement scale.

## Discussion

This paper reviewed studies reporting the prognosis of CFS following CBT and GET. The review included 15 publications, comprising 11 studies of CBT and six of GET. Results, implications, and limitations will now be discussed.

### Prognosis following CBT

Findings from the synthesis revealed that, at post-treatment to short-term follow-up, fatigue significantly improved in 43% of participants, 23% more than in control conditions. These results are consistent with Price et al. (2008) who found that 40–48% of participants showed clinically significant post-treatment improvements in fatigue, significantly more than in control groups. Findings also showed that, at short-term to medium-term follow-up, 44% of participants considered themselves significantly improved, 20% more than in control groups, and 59% demonstrated clinically significant improvements in physical functioning, 14% more than in control groups. These results are in line with the positive effects of CBT at short-term to medium-term follow-up, although further work is needed to build on these given the inconsistent findings of previous reviews. For example, Price et al. (2008) found that, at short-term to medium-term follow-up, CBT led to significant improvements in physical functioning compared to other treatments such as relaxation and counselling, but not compared to treatment as usual. Finally, the synthesis revealed that, at short-term to medium-term follow-up, 11% of participants considered themselves worse, 8% fewer than in control conditions.

### Prognosis following GET

Findings from the data synthesis revealed that at post-treatment to medium-term follow-up, 61% of participants demonstrated clinically significant improvements in physical functioning, 23% more than in control conditions, and at post-treatment to short-term follow-up, 43% considered themselves significantly better, 26% more than in control conditions. These findings are consistent with a previous review of GET (Larun et al., 2019) which reported that treatment may moderately improve physical functioning and increase the number of people who report at least some improvement at post-treatment, although findings regarding outcomes at short, medium, and long-term are mixed, and based on fewer studies.

The synthesis also showed that, at post-treatment to short-term follow-up, 14% of participants considered themselves worse, 10% fewer than in control groups. This is consistent with a review of GET (White & Etherington, 2021) which found no evidence of excess harm with GET by either self-rated deterioration or by withdrawing, although their finding that more GET participants dropped out of trial follow-up in comparison with control interventions requires further investigation.

### Clinical implications

Our analysis indicates that, at post-treatment to medium-term follow-up, CBT and GET result in a better prognosis compared to control conditions. These findings are consistent with previous reviews reporting post-treatment outcomes and may provide some support for the positive effects of CBT and GET at short-term to medium-term follow-up, although this requires further investigation given that a meta-analysis was not conducted and the inconsistent findings of previous reviews. Nonetheless, our findings are consistent with previous findings that CBT and GET yield similar improvements in fatigue and functioning. Commissioners may want to consider this when providing treatment.

### Limitations

A limitation of this review is that one-third of included studies were of weak quality. All studies were penalized for a lack of blinding, which increases the risk of performance or detection bias. However, blinding is not possible in trials of psychotherapeutic treatments while it has been suggested that concerns over bias caused by lack of blinding may be exaggerated (Moustgaard et al., 2020). Five CBT studies were penalized because drop-outs were not adequately described, which can indicate a vulnerability to attrition bias. However, when weak studies were excluded from the analysis, estimates of improvement in fatigue and functioning following CBT remained similar, while all studies used in the synthesis of global improvement and worsening, as well all GET studies, were of moderate quality. This is consistent with previous reviews of CBT and GET which found that the quality of methodological features such as attrition and blinding do not predict effect size for improvements in fatigue and physical functioning (Castell et al., 2011; Malouff et al., 2008; Marques et al., 2015). This suggests that the inclusion of studies rated as weak may not significantly affect estimates of prognosis in the current review.

Another limitation relates to the clinical heterogeneity of included studies, which used different diagnostic criteria, investigated different intensities and doses of treatments, and assessed outcomes at different follow-ups. This may have contributed to between-study differences in prognosis. Indeed, even though they are similar, studies using Oxford criteria for CFS may yield non-significantly higher effect sizes than those using CDC criteria (Castell et al., 2011; Larun et al., 2019). However, all included studies investigated CBT or GET which are underpinned by a bio-psycho-social model of CFS, increasing their comparability. Another contributor to heterogeneity relates to the different thresholds used by study authors to determine improvements in physical functioning following both CBT and GET, and this outcome should therefore be interpreted with caution. The same absolute cut-off scores were used by study authors to calculate improvements in fatigue following CBT, although the reliable change index was used inconsistently as additional criteria and this outcome should therefore also be interpreted with caution.

A further limitation is that most studies reported subjective, self-report measures, which may have increased the risk of observer or detection bias. However, two included studies (Jason et al., 2007; Sharpe et al., 1996) reported that CBT yielded greater increases in employment than did control conditions. Nonetheless, other research (Deale, Husain, Chalder, & Wessely, 2001; McCrone et al., 2012) has found only modest increases in employment, while subjective and objective measures do not necessarily correlate (King, Beynon, Chalder, Sharpe, & White, 2020). These findings suggest that future trials should obtain a range of outcome measures and investigate potential discrepancies between them.

The risk of review-level bias, and missing relevant articles, was minimized by using four databases to search the literature, in addition to manual and reference list searches. Publication bias was not assessed, and although Castell et al. (2011) found little difference between the effect sizes of published and unpublished CBT trials, Marques et al. (2015) found some evidence of reporting bias for fatigue and physical functioning in their review of interventions with a graded exercise component. Whilst the extent of reporting bias in the current review is unclear, the potential for this bias was minimized by extracting dichotomized outcomes regardless of whether they pertained to the primary outcome analysis, in addition to obtaining unpublished data relating to global worsening in three studies included in the data synthesis.

A final limitation is that treatment in most trials involved at least some face-to-face sessions, thereby excluding less mobile or housebound participants, therefore findings may not be generalizable to severe CFS.

### Future directions

Future trials should report objective outcomes, in addition to self-report measures, which are essential as fatigue is subjective and individuals are best placed to judge how they feel. Further work also needs to investigate the statistical significance of between-treatment differences in prognosis, particularly in the longer term, and whether differences are moderated by diagnostic criteria and treatment intensity. There is a need to further define and measure deterioration and recovery. Further trials, not only of CBT and GET, but also other treatments, are required, particularly for individuals with severe CFS.

## Conclusions

Findings of this review indicate that CBT and GET lead to a more favorable prognosis compared to control groups comprising relaxation, medical care, and wait-list conditions. These findings correspond with existing reviews reporting improvements in post-treatment symptoms; however, previous findings concerning short-term to medium-term follow-up are inconsistent and require further investigation. Further work also needs to improve existing treatments and to explore new treatments, particularly for those with severe CFS.

## Supplementary material

For supplementary material accompanying this paper visit https://doi.org/10.1017/S0033291722002471. click here to view supplementary material
