# Supplementary material for: A systematic literature review of randomized controlled trials evaluating prognosis following treatment for adults with chronic fatigue syndrome
Source: Psychol Med. 2022 Sep 5;52(14):2917–29. doi: 10.1017/S0033291722002471 (PMC9693680; doi:10.1017/S0033291722002471)
Supplement: Supplementary file 1 [file S0033291722002471sup.zip › S0033291722002471sup001.docx]

## eAppendix 1: Search criteria

1. MEDLINE, EMBASE, PsycINFO:

1. (chronic fatigue syndrome or chronic fatigue or myalg* encephal* or asthenia or neurasthenia or post?viral fatigue* or ME?CFS or systematic exertion intolerance disease.ti,ab,kw.
2. (cognitive behavio?ral therapy or CBT or cognitive therapy or behavio* or behavio?al or graded exercise* or GET or drug or medicine or pharmaco* or occupational therapy or pragmatic rehabilitation or rebahabilit* or complementary* or treatment* or intervention*).ti,ab,kw.
3. randomized controlled trial.pt.
4. controlled clinical trial.pt.
5. randomi?ed.ab.
6. placebo.ab.
7. randomly.ab.
8. trial.ab.
9. groups.ab.
10. 3 or 4 or 5 or 6 or 7 or 8 or 9
11. (outcome or recovery or prognosis or improvement or fatigue or physical functioning or functioning or "quality adj2 life" or "work adj3 social adjustment scale" or predict* or moderat* or correlat* or mediat* or association*).ti,ab,kw.
12. 1 and 2 and 10 and 11
13. (child* or adolscen*).ti,ab,kw.
14. 12 not 13
15. Limit to human
16. Limit to 1988-present

**2. Web of Science:**

1. ("chronic fatigue syndrome" or “chronic fatigue” or "myalg* encephal*" or "asthenia" or "neurasthenia" or “post?viral fatigue*” or “ME?CFS” or “systemic exertion intolerance disease”).
2. ("cognitive behavio?ral therapy" or "CBT" or "behavio*" or “behavio?al” or "cognitive therapy" or "graded exercise*" or "GET" or "drug" or "medicine" or "pharmaco*" or "occupational therapy" or "pragmatic rehabilitation" or "rehabilit*" or "complementary*" or "treatment*" or "intervention*")
3. (“randomi?ed controlled trial” or “controlled clinical trial” or “randomi?ed” or “placebo” or “randomly” or “trial” or “groups”)
4. (“outcome” or “recovery” or “prognosis” or “improvement” or “fatigue” or “functioning” or “physical functioning” or “quality adj2 life” or “work adj3 social adjustment scale” or “predict*” or “moderat*” or “correlat*” or “mediat*” or “association*”)
5. **Not** ("child*" or "adolescen*")
