# Supplementary material for: A systematic literature review of randomized controlled trials evaluating prognosis following treatment for adults with chronic fatigue syndrome
Source: Psychol Med. 2022 Sep 5;52(14):2917–29. doi: 10.1017/S0033291722002471 (PMC9693680; doi:10.1017/S0033291722002471)
Supplement: Supplementary file 1 [file S0033291722002471sup.zip › S0033291722002471sup003.docx]

**eAppendix 3: Excluded studies**

As summarized in Figure 1, the current review excluded 35 studies for the following reasons.

- Ten studies were excluded because they investigated multidisciplinary treatments, combination treatments, or because treatment conditions were pooled in the analysis (Brown, Khorana, & Jason, 2011; Goldsmith, Dunn, Bentall, Lewis, & Wearden, 2015; Hlavaty, Brown, & Jason, 2011; Jason, Benton, & Torres-Harding, 2009; Lloyd et al., 1993; Nunez et al., 2011; Wearden et al., 2010; Wearden, Dunn, Dowrick, & Morriss, 2012; Wearden et al., 1998; Wearden & Emsley, 2013).
- Eight studies were excluded as they were not randomized or controlled (Powell et al., 2004; Quarmby et al., 2007; Roberts et al., 2010; Sharpe, 1998; Thomas et al., 2006; Thomas et al., 2008; Thomas & Smith, 2007; White & Naish, 2001).
- Six studies were excluded because they did not meet our criteria for CBT or GET in either content or duration, or both (Hall et al., 2017; Lopez et al., 2011; Pinxsterhuis, Sandvik, Strand, Bautz-Holter, & Sveen, 2017; Rimes & Wingrove, 2013; Santaella, Font, & Disdier, 2004; Taylor, 2004).
- Five studies were excluded because screening or assessment of CFS was not conducted by a secondary care medical doctor or psychiatrist (O’Dowd, Gladwell, Rogers, Hollinghurst, & Gregory, 2006; Tummers, Knoop, van Dam, & Bleijenberg, 2012, 2013; Wallman, Morton, Goodman, Grove, & Guilfoyle, 2004; Whitehead & Campion, 2002).
- Two studies were excluded as participants did not meet full criteria for CFS (Prins et al., 2001; Prins, Bleijenberg, Rouweler, & van der Meer, 2005).
- One study was excluded as it did not report original data (Wilshire, Kindlon, Matthees, & McGrath, 2017).
- One study was excluded as it did not report dichotomous outcomes of interest (Burgess, Andiappan, & Chalder, 2012).
- One study was excluded as a diagnosis of idiopathic chronic fatigue was used as entry criteria (Prins, Bazelmans, van der Werf, van der Meer, & Bleijenberg, 2002).
- One study was excluded as participants had received non-study treatment (Deale, Husain, Chalder, & Wessely, 2001).

**References**

Brown, M., Khorana, N., & Jason, L. (2011). The Role of Changes in Activity as a Function of Perceived Available and Expended Energy in Nonpharmacological Treatment Outcomes for ME/CFS. *Journal of Clinical Psychology*, *67*(3), 253–260. https://doi.org/10.1002/jclp.20744

Burgess, M., Andiappan, M., & Chalder, T. (2012). Cognitive behaviour therapy for chronic fatigue syndrome in adults: face to face versus telephone treatment: a randomized controlled trial. *Behavioural and Cognitive Psychotherapy*, *40*(2), 175–191. https://doi.org/10.1017/s1352465811000543

Deale, A., Husain, K., Chalder, T., & Wessely, S. (2001). Long-term outcome of cognitive behavior therapy versus relaxation therapy for chronic fatigue syndrome: A 5-year follow-up study. *American Journal of Psychiatry*, *158*(12), 2038–2042. https://doi.org/10.1176/appi.ajp.158.12.2038

Goldsmith, L. P., Dunn, G., Bentall, R. P., Lewis, S. W., & Wearden, A. J. (2015). Therapist Effects and the Impact of Early Therapeutic Alliance on Symptomatic Outcome in Chronic Fatigue Syndrome. *PloS One*, *10*(12). https://doi.org/10.1371/journal.pone.0144623

Hall, D. L., Lattie, E. G., Milrad, S. F., Czaja, S., Fletcher, M. A., Klimas, N., … Antoni, M. H. (2017). Telephone-administered versus live group cognitive behavioral stress management for adults with CFS. *Journal of Psychosomatic Research*, *93*(pp 41-47). Retrieved from https://openathens.ovid.com/secure-ssl/home.oa?idpselect=https://kclidp.kcl.ac.uk/idp/shibboleth&entityID=https://kclidp.kcl.ac.uk/idp/shibboleth&?T=JS&CSC=Y&NEWS=N&PAGE=fulltext&D=emexa&AN=613702967

Hlavaty, L., Brown, M., & Jason, L. (2011). The effect of homework compliance on treatment outcomes for participants with myalgic encephalomyelitis/chronic fatigue syndrome. *Rehabilitation Psychology*, *56*(3), 212–218. https://doi.org/10.1037/a0024118

Jason, L. A., Benton, M., & Torres-Harding, S. (2009). The impact of energy modulation on physical functioning and fatigue severity among patients with ME/CFS. *Patient Educ Couns.*, *77*(2), 237–241.

Lloyd, A. R., Hickie, I., Brockman, A., Hickie, C., Wilson, A., Dwyer, J., & Wakefield, D. (1993). Immunologic and psychologic therapy for patients with chronic fatigue syndrome: A double-blind, placebo-controlled trial. *The American Journal of Medicine*, *94*(2), 197–203. https://doi.org/10.1016/0002-9343(93)90183-P

Lopez, C., Antoni, M., Penedo, F., Weiss, D., Cruess, S., Segotas, M. C., … Fletcher, M. A. (2011). A pilot study of cognitive behavioral stress management effects on stress, quality of life, and symptoms in persons with chronic fatigue syndrome. *Journal of Psychosomatic Research*, *70*(4), 328–334. https://doi.org/10.1016/j.jpsychores.2010.11.010

Nunez, M., Fernandez-Sola, J., Nunez, E., Fernandez-Huerta, J. M., Godas-Sieso, T., & Gomez-Gil, E. (2011). Health-related quality of life in patients with chronic fatigue syndrome: group cognitive behavioural therapy and graded exercise versus usual treatment. A randomised controlled trial with 1 year of follow-up. *Clinical Rheumatology*, *30*(3), 381–389. https://doi.org/10.1007/s10067-010-1677-y

O’Dowd, H., Gladwell, P., Rogers, C. A., Hollinghurst, S., & Gregory, A. (2006). Cognitive behavioural therapy in chronic fatigue syndrome: a randomised controlled trial of an outpatient group programme. *Health Technology Assessment*, *10*(37), 1–121. https://doi.org/10.3310/hta10370.

Pinxsterhuis, I., Sandvik, L., Strand, E. B., Bautz-Holter, E., & Sveen, U. (2017). Effectiveness of a group-based self-management program for people with chronic fatigue syndrome: a randomized controlled trial. *Clinical Rehabilitation*, *31*(1), 93–103. https://doi.org/10.1177/0269215515621362

Powell, P., Bentall, R. P., Nye, F. J., & Edwards, R. H. (2004). Patient education to encourage graded exercise in chronic fatigue syndrome. 2-year follow-up of randomised controlled trial. *British Journal of Psychiatry*, *184*, 142–146. Retrieved from https://openathens.ovid.com/secure-ssl/home.oa?idpselect=https://kclidp.kcl.ac.uk/idp/shibboleth&entityID=https://kclidp.kcl.ac.uk/idp/shibboleth&?T=JS&CSC=Y&NEWS=N&PAGE=fulltext&D=med5&AN=14754826

Prins, J. B., Bazelmans, E., van der Werf, S., van der Meer, J. W. M., & Bleijenberg, G. (2002). Cognitive behaviour therapy for chronic fatigue syndrome: predictors of treatment outcome. *Psycho-Neuro-Endocrino-Immunology*, *1241*, 131–135. https://doi.org/10.1016/S0531-5131(02)00632-5

Prins, J. B., Bleijenberg, G., Bazelmans, E., Elving, L. D., de Boo, T. M., Severens, J. L., … van der Meer, J. W. (2001). Cognitive behaviour therapy for chronic fatigue syndrome: a multicentre randomised controlled trial. *Lancet*, *357*(9259), 841–847. https://doi.org/10.1016/S0140-6736(00)04198-2

Prins, J. B., Bleijenberg, G., Rouweler, E. F., & van der Meer, J. (2005). Effect of psychiatric disorders on outcome of cognitive-behavioural therapy for chronic fatigue syndrome. *British Journal of Psychiatry*, *187*(2), 184–185. https://doi.org/10.1192/bjp.187.2.184

Quarmby, L., Rimes, K. A., Deale, A., Wessely, S., & Chalder, T. (2007). Cognitive-behaviour therapy for chronic fatigue syndrome: Comparison of outcomes within and outside the confines of a randomised controlled trial. *Behaviour Research and Therapy*, *45*(6), 1085–1094. https://doi.org/10.1016/j.brat.2006.08.019

Rimes, K. A., & Wingrove, J. (2013). Mindfulness-Based Cognitive Therapy for People with Chronic Fatigue Syndrome Still Experiencing Excessive Fatigue after Cognitive Behaviour Therapy: A Pilot Randomized Study. *Clinical Psychology & Psychotherapy*, *20*(2), 107–117. https://doi.org/10.1002/cpp.793

Roberts, A. D. L., Charler, M. L., Papadopoulos, A., Wessely, S., Chalder, T., & Cleare, A. J. (2010). Does hypocortisolism predict a poor response to cognitive behavioural therapy in chronic fatigue syndrome? *Psychological Medicine*, *40*(3), 515–522. https://doi.org/10.1017/S0033291709990390

Santaella, M. L., Font, I., & Disdier, O. M. (2004). Comparison of oral nicotinamide adenine dinucleotide (NADH) versus conventional therapy for chronic fatigue syndrome. *Puerto Rico Health Sciences Journal*, *23*(2), 89–93. Retrieved from https://openathens.ovid.com/secure-ssl/home.oa?idpselect=https://kclidp.kcl.ac.uk/idp/shibboleth&entityID=https://kclidp.kcl.ac.uk/idp/shibboleth&?T=JS&CSC=Y&NEWS=N&PAGE=fulltext&D=emed9&AN=39352601

Sharpe, M. (1998). Cognitive behavior therapy for chronic fatigue syndrome: Efficacy and implications. *American Journal of Medicine*, *105*(3 A), 104S-109S. https://doi.org/10.1016/s0002-9343(98)00170-3

Taylor, R. R. (2004). Quality of life and symptom severity for individuals with chronic fatigue syndrome: Findings from a randomized clinical trial. *American Journal of Occupational Therapy*, *58*(1), 35–43. https://doi.org/10.5014/ajot.58.1.35

Thomas, M. A., Sadlier, M. J., & Smith, A. P. (2008). A multiconvergent approach to the rehabilitation of patients with chronic fatigue syndrome: a comparative study. *Physiotherapy*, *94*(1), 35–42. https://doi.org/10.1016/j.physio.2007.04.013

Thomas, M., Sadlier, M., & Smith, A. (2006). The effect of Multi Convergent Therapy on the psychopathology, mood and performance of Chronic Fatigue Syndrome patients: A preliminary study. *Counselling & Psychotherapy Research*, *6*(2), 91–99. Retrieved from https://openathens.ovid.com/secure-ssl/home.oa?idpselect=https://kclidp.kcl.ac.uk/idp/shibboleth&entityID=https://kclidp.kcl.ac.uk/idp/shibboleth&?T=JS&CSC=Y&NEWS=N&PAGE=fulltext&D=psyc5&AN=2006-12420-002

Thomas, M., & Smith, A. (2007). An evaluation of counselling and rehabilitation courses for Chronic Fatigue Syndrome. *Counselling & Psychotherapy Research*, *7*(3), 164–171. Retrieved from https://openathens.ovid.com/secure-ssl/home.oa?idpselect=https://kclidp.kcl.ac.uk/idp/shibboleth&entityID=https://kclidp.kcl.ac.uk/idp/shibboleth&?T=JS&CSC=Y&NEWS=N&PAGE=fulltext&D=psyc5&AN=2007-12867-006

Tummers, M., Knoop, H., van Dam, A., & Bleijenberg, G. (2012). Implementing a minimal intervention for chronic fatigue syndrome in a mental health centre: a randomized controlled trial. *Psychological Medicine*, *42*(10), 2205–2215. https://doi.org/10.1017/S0033291712000232

Tummers, M., Knoop, H., van Dam, A., & Bleijenberg, G. (2013). Moderators of the treatment response to guided self-instruction for chronic fatigue syndrome. *Journal of Psychosomatic Research*, *74*(5), 373–377. https://doi.org/10.1016/j.jpsychores.2013.01.007

Wallman, K. E., Morton, A. R., Goodman, C., Grove, R., & Guilfoyle, A. M. (2004). Randomised controlled trial of graded exercise in chronic fatigue syndrome. *Medical Journal of Australia*, *180*(9), 444–448. https://doi.org/10.5694/j.1326-5377.2004.tb06019.x

Wearden, A. J., Dowrick, C., Chew-Graham, C., Bentall, R. P., Morriss, R. K., Peters, S., … Dunn, G. (2010). Nurse led, home based self help treatment for patients in primary care with chronic fatigue syndrome: randomised controlled trial. *BMJ*, *340*(1777), 1–12. https://doi.org/10.1136/bmj.c1777

Wearden, A. J., Dunn, G., Dowrick, C., & Morriss, R. K. (2012). Depressive symptoms and pragmatic rehabilitation for chronic fatigue syndrome. *British Journal of Psychiatry*, *201*(3), 227–232. https://doi.org/10.1192/bjp.bp.111.107474

Wearden, A. J., & Emsley, R. (2013). Mediators of the Effects on Fatigue of Pragmatic Rehabilitation for Chronic Fatigue Syndrome. *Journal of Consulting and Clinical Psychology*, *81*(5), 831–838. https://doi.org/10.1037/a0033561

Wearden, A. J., Morriss, R. K., Mullis, R., Strickland, P. L., Pearson, D. J., Appleby, L., … Morris, J. A. (1998). Randomised, double-blind, placebo-controlled treatment trial of fluoxetine and graded exercise for chronic fatigue syndrome. *British Journal of Psychiatry*, *172*, 485–490. https://doi.org/10.1192/bjp.172.6.485

White, P. D., & Naish, V. A. (2001). Graded exercise therapy for chronic fatigue syndrome. *Physiotherapy*, *87*(6), 285–288. https://doi.org/10.1016/S0031-9406(05)60762-6

Whitehead, L., & Campion, P. (2002). Can general practitioners manage Chronic Fatigue Syndrome? A controlled trial. *Journal of Chronic Fatigue Syndrome*, *10*(1), 55–64. Retrieved from https://openathens.ovid.com/secure-ssl/home.oa?idpselect=https://kclidp.kcl.ac.uk/idp/shibboleth&entityID=https://kclidp.kcl.ac.uk/idp/shibboleth&?T=JS&CSC=Y&NEWS=N&PAGE=fulltext&D=emed8&AN=35205035

Wilshire, C., Kindlon, T., Matthees, A., & McGrath, S. (2017). Can patients with chronic fatigue syndrome really recover after graded exercise or cognitive behavioural therapy? A critical commentary and preliminary re-analysis of the PACE trial. *Fatigue-Biomedicine Health and Behavior*, *5*(1), 43–56. https://doi.org/10.1080/21641846.2017.1259724
